# Virucidal Activity of Gold Nanoparticles Synthesized by Green Chemistry Using Garlic Extract

**DOI:** 10.3390/v11121111

**Published:** 2019-11-30

**Authors:** Mayra A. Meléndez-Villanueva, Karla Morán-Santibañez, Juan J. Martínez-Sanmiguel, Raúl Rangel-López, Marco A. Garza-Navarro, Cristina Rodríguez-Padilla, Diana G. Zarate-Triviño, Laura M. Trejo-Ávila

**Affiliations:** 1 Facultad de Ciencias Biológicas, Universidad Autónoma de Nuevo León, Ciudad Universitaria, CP 66455 San Nicolás de los Garza, Mexico; mayra.melendez@hotmail.com (M.A.M.-V.); karla.moransn@uanl.edu.mx (K.M.-S.); jrangelraul95@gmail.com (R.R.-L.); crrodrig07@gmail.com (C.R.-P.); 2 Facultad de Ingeniería Mecánica y Eléctrica, Universidad Autónoma de Nuevo León, Ciudad Universitaria, CP 66455 San Nicolás de los Garza, Mexico; ingmarcogarza@gmail.com

**Keywords:** measles, antivirals, gold nanoparticles, virucidal activity

## Abstract

Measles virus (MeV) is a paramyxovirus that infects humans, principally children. Despite the existence of an effective and safe vaccine, the number of cases of measles has increased due to lack of vaccination coverage. The World Health Organization (WHO) reports that the number of cases worldwide multiplied fourfold between January and March 2019, to 112,000. Today, there is no treatment available for MeV. In recent years, it has been demonstrated that natural extracts (herbal or algal) with antiviral activity can also work as reducing agents that, in combination with nanotechnology, offer an innovative option to counteract viral infections. Here, we synthetized and evaluated the antiviral activity of gold nanoparticles using garlic extract (*Allium sativa*) as a reducing agent (AuNPs-As). These nanoparticles actively inhibited MeV replication in Vero cells at a 50% effective concentration (EC_50_) of 8.829 µg/mL, and the selectivity index (SI) obtained was 16.05. AuNPs-As likely inhibit viral infection by blocking viral particles directly, showing a potent virucidal effect. Gold nanoparticles may be useful as a promising strategy for treating and controlling the infection of MeV and other related enveloped viruses.

## 1. Introduction

Measles is a viral disease characterized by fever, skin rash, cough and conjunctivitis, and a generalized immune suppression. The disease is caused by an enveloped virus with a single-strand RNA negative polarity ssRNA (−) genome belonging to the family [1]. Measles can lead to opportunistic infections and complications, such as pneumonia and gastro-intestinal diseases, due to increased susceptibility. Safe and effective, live-attenuated virus vaccines have led to a substantial reduction in measles morbidity and mortality [2], although, in recent years the number of cases and deaths has increased. Measles resurgence may be imminent thanks to reduced measles vaccine acceptance in the industrialized world and the lack of vaccination coverage in developing countries [3]. The World Health Organization (WHO) estimated that approximately 114,900 people, mostly children under five years of age, died of measles and resulting sequelae in 2014 [4].

Despite significant achievements in the search for new antiviral treatments, the number of available antiviral compounds remains limited. Current antivirals are only effective against a select group of pathogens, as well, and notably, there is no antiviral drugs currently available against Measles virus (MeV) [5].

Recently, it has been reported the use of natural bioactive compounds, as reducing agents of metal salts in the reduction of silver and gold ions to Nanoparticles (NPs) with antiviral properties [6]. The synthesis of NPs by green chemistry provides an advance due to is cost-effective and environment-friendly characteristics, easy to apply for large scale synthesis, and in this method, there is no need to use some techniques, including high pressure, temperature, or toxic chemicals [7]. 

In this study, gold nanoparticles using a garlic extract (AuNPs-As) as a reducing agent were synthesized and the antiviral activity against MeV was evaluated. Nanoparticles have been proposed as antiviral systems, because they show activity against enveloped and non-enveloped viruses. Today, nanotechnology offers an innovative option to counteract viral infections. Different mechanisms of action have been described, inside (in replication process, translation, transcription) or outside the cell (blocking the entry or exit of the virions) [8]. At the present time, the antibacterial, anticancer and antioxidant properties of nanoparticles have been widely explored, however, their antiviral properties remain an area in development. Hence, we propose the use of nanoparticles as an antiviral alternative for therapeutic use against MeV.

## 2. Materials and Methods 

### 2.1. Preparation of Garlic (Allium sp.) Extract

Two hundred grams of garlic bulbs were peeled and disinfected with a 1% chlorine solution for 5 min, and then the garlic was crushed and boiled at 100 °C for 5 min in 500 mL distilled water. Afterward, the mixture was filtered. The final product was lyophilized for 24 h. 

### 2.2. Synthesis of Gold Nanoparticles

Gold nanoparticles (AuNPs) were obtained according to the Turkevich method [9] which was modified using garlic extract (As) as reducing agent. One hundred to one of a solution 0.1 mg/mL of garlic extract was added to a 1 mL of 1 mM HAuCl_4_ (St. Louis, MO, USA). The pH of the resulting solution was adjusted with NaOH at 1 N until reaching a pH of 9, and then the sample was heated to 100 ± 5 °C with constant agitation for 20 min. A variation of yellow to red indicates the formation of gold nanoparticles reduced with garlic extract (AuNPs-As), and finally, the AuNPs-As was passed through 0.2 µm filter.

### 2.3. Characterization of AuNPs-As

The characteristic resonance plasmon of nanoparticles was measured by UV-Vis absorption spectroscopy equipment (NanoDrop 2000 Spectrophotometer, Thermo Scientific^®^) with a frequency range between 300 to 700 nm. The size and Z potential of the AuNPs-As was determined by dynamic light scattering (DLS) using a nanosizer NS90 (Malvern Instruments, Malvern, UK). The morphology and size of gold nanoparticles, as well as its interaction with the virus, were observed by transmission electron microscopy (TEM) in a Field Emission Gun, FEI TITAN G2 80-300, operated at 300 kV, using techniques such as bright field (BF) and Z-contrast (HAADF-STEM) imaging.

### 2.4. Cell Culture and Virus Propagation

Vero cells (ATCC^®^ CCL-81™) were cultured at 37 °C in a 5% CO_2_ atmosphere in Dulbecco’s Modified Eagle Medium Nutrient Mixture F-12 (DMEM/F12, Gibco Invitrogen, Gaithersburg, MD, USA) supplemented with 5% fetal bovine serum (FBS, Gibco Invitrogen, Gaithersburg, MD, USA) and 1% antibiotic (Gibco Invitrogen, Gaithersburg, MD, USA).

Measles virus (Edmoston strain) was purchased from ATCC (ATCC^®^ VR-24™). The virus was propagated on Vero cells and viral titers were determined by Plaque Formation Units. Aliquots of viral stock were stored at −30 °C until use.

### 2.5. Viability Assays

The effect of AuNPs-As and their precursors, HAuCl_4_ and garlic extract (*Allium sativum*) on Vero cells viability was determined by MTT assay (3-[4,5-dimethylthiazole-2-yl]-2,5-diphenyltetrazolium bromide) [10]. The cells were cultured in 96-well plates at a density of 15,000 cells/well at 37 °C in an atmosphere of 5% CO_2_ until confluence of 80–90%. Several concentrations (0.1, 1, 10, 100, and 250 µg/mL) of treatments diluted in DMEM were added; after 48 h at 37 °C and 5% of CO_2_ of incubation, the media were replaced with 22 µL of 5 mg/mL MTT dissolved in phosphate-buffered saline (PBS). After 2 h, 100 µL of DMSO was added. The optical density at a wavelength of 450 nm (OD450 nm) was measured using a microplate reader (Multiskan FC, Thermo Fisher Scientific, Bedford, MA, USA). Cell viability was expressed by percentage as the mean value of three independent experiments considering control cells absorbance as 100% viable. CC_50_ was the concentration of the test substances that inhibited the Vero cells growth by 50%, compared with the growth of the untreated cells.

### 2.6. Antiviral Activity Assay

The cells were cultured in 6-well plates at a density of 320,000 cells/well at 37 °C in an atmosphere of 5% CO_2_ until an 80–90% confluence. Vero cells were infected with 80 to 90 Plaque Formation Units (PFU) of MeV and treatments AuNPs-As and their precursors, HAuCl_4_ and garlic extract (*Allium sativum*) at different concentrations (0.03–10 μg/mL), virus and treatments were added in a volume of 1 mL, for a total of 2 mL per well. After 1 h at 37 °C and 5% of CO_2_ of incubation, the viral inoculum and treatment were removed, and the monolayer was overlaid with medium containing 0.6% agar (MP Biomedicals, Cat No. 194615 Lot No. R19854) in 2 mL of DMEM. After 72 h of incubation in a controlled environment, the agar layer was removed and fixed with methanol-acetone 1:1 for 30 min at −30 °C and stained with 1% crystal violet. The values of effective concentration by 50% (EC_50_) were determined by regression model, and SI was calculated with values of CC_50_ previously obtained with the formula below. The evaluation of treatments was achieved using three wells with each concentration evaluated. This trail was done in triplicate.

$$SI=\frac{CC50\%}{EC50\%}.$$

### 2.7. Time-of-Addition Assay

Vero cell monolayers were infected with 80–90 PFUs of MeV. NPs was added at a concentration of 8.829 μg/mL (EC_50_) at different times of infection: 60 min pre-infection, 0, 15, 30 min, 1, 2, 4, and 6 h post-infection. Thereafter, for each treatment, cells were incubated with NPs for 1 h and then washed with PBS, the monolayer was overlaid with medium containing 0.6% agar (MP Biomedicals) in 2 mL of DMEM; after 72 h of incubation in controlled environment, the agar layer was removed and fixed with methanol-acetone 1:1 for 30 min at −30 °C and stained with 1% crystal violet; subsequently, plaques were counted. This trial was done in triplicate.

### 2.8. Virucidal Assay 

The virucidal activity of AuNPs-As against MeV was assessed using plaque reduction assay. The Vero cells were cultured in 6-well plates at a density of 320,000 cells/well at 37 °C in an atmosphere of 5% CO_2_ until an 80–90% confluence; old medium was removed. The assays were performed by adding 500 μL of MeV in a density of 80–90 PFU and 500 μL of NPs for 0, 3, and 6 h at a concentration of EC_50_. The plate was incubated 1 h at 37 °C and 5% of CO_2_, mix of NPs, and virus was removed and was overlaid with medium containing 0.6% agar (MP Biomedicals) in 2 mL of DMEM; after 72 h of incubation on controlled environment, agar was removed and fixed with methanol-acetone 1:1 for 30 min at −30 °C and stained with 1% crystal violet. The PFU were counted. This trial was done in triplicate. 

### 2.9. RT-qPCR

Quantitative Real-Time PCR was performed, total RNA was isolated from treated Vero cells using TRIzol™ Reagent Invitrogen™ (Thermo Fisher Scientific, Bedford, MA, USA). Reverse transcription was carried out using the High Capacity cDNA Reverse Transcription Kit (Applied Biosystems, Beverly, MA, USA) and the viral genome was amplified with the specific primers: MeVF: 5′ GAGGGTCAAACAGAGTCGAG 3′, MeVR: 5′ CGGTTGGAAGATGGGCAG 3′ that amplified a 95 nt fragment [11]. The real-time PCR was carried out using PowerUp™ SYBR™ Green Master Mix (Applied Biosystems, Beverly, MA, USA) and the Applied Biosystems StepOne Real Time PCR System following procedures: 95 °C for 2 min, followed by 40 cycles of 95 °C for 2 s, 60 °C for 10 s, and 72 °C for 20 s. The number of viral copies was calculated by using a standard curve. Serial 10-fold dilutions of a synthetic oligonucleotide encompassing the target measles gene were used to establish the standard curves.

### 2.10. Statistical Analysis 

The CC_50_ and EC_50_ values were calculated using GraphPad prism 7.0 software (GraphPad Software, Inc., San Diego, CA, USA). All data were expressed as mean standard deviation (SD). All experiments were performed in triplicate. The results were submitted to one way and two-way ANOVA test using GraphPad prism 7.0.

## 3. Results

### 3.1. Characterization of AuNPs-As

**Figure 1:**
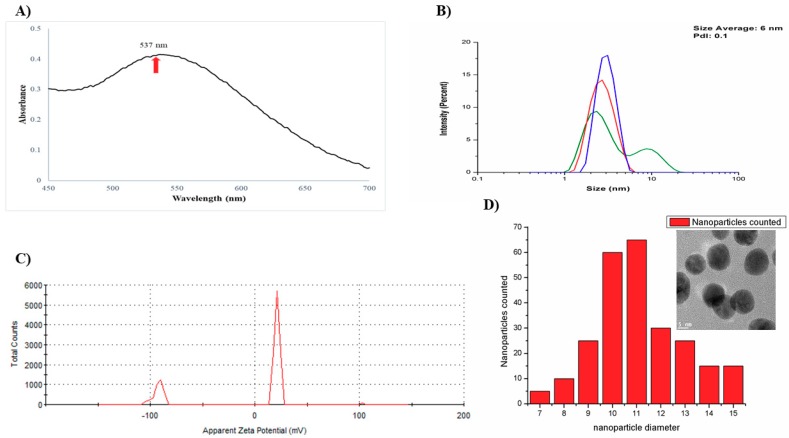
Structure and characterization of gold nanoparticles using a garlic extract (AuNPs-As). (**A**) Surface plasmon absorption of AuNPs-As. (**B**) Size distribution of the AuNPs-As, as measured by dynamic light scattering (DLS). (**C**) Surface charge measured by Z potential. (**D**) Histogram of AuNPs-As size and TEM image of the AuNPs-As.

The ultraviolet-visible spectroscopy of AuNPs-As showed a characteristic resonance plasmon at 537 nm (Figure 1A), and two nanoparticles populations were observed by DLS (Figure 1B) the average size was of 6 nm with a low polydispersity index (PdI), indicating a low variation in size distribution. The zeta potential was of +21.2 mV, as Figure 1C shows. In addition, the histogram of particle size distribution from TEM obtained was 11 nm. (Figure 1D). 

### 3.2. Cytotoxicity of AuNPs

**Figure 2:**
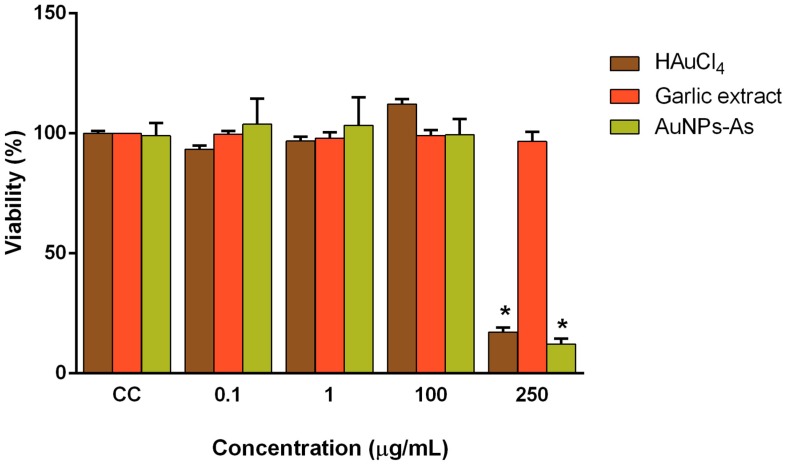
Viability of Vero cells in the presence of AuNPs-As, HAuCl_4_, and garlic extract. Vero cells (1.5 × 10^4^ cells) plated in 96-well plates were treated with different concentrations of AuNPs-As during 72 h and the viability was determined with an MTT assay. The data are expressed as relative viability (%) to that for the untreated control cells, which was defined as 100%. The data shown are the mean ± SD from three replicated experiments. α ≤ 0.05 (*).

Vero cells (which express CD46 receptor) were used as a model to assess gold nanoparticles cytotoxicity. The toxicity of AuNPs-As and their precursors, HAuCl_4_ and garlic extract (*Allium sativum*) was assessed at different concentrations by colorimetric MTT assay (Figure 2). A significant viability reduction was obtained only with the highest concentration tested (200 μg/mL) of AuNPs-As and HAuCl_4_. The cytotoxicity concentration 50% (CC_50_) was determined at 141.75 µg/mL for AuNPs-As and 231.74 μg/mL for HAuCl_4_. We also performed a viability assay using gold nanoparticles synthesized by the technique previously reported by Martínez-Torres et al. [12] and using chitosan as reducing agent (AuNPs-Chi) to compare the effect of both techniques [13]. This synthesis showed a cytotoxicity of 68.86% at a concentration of 10 μg/mL, which is higher than that of AuNPs-As.

### 3.3. Antiviral Activity 

**Figure 3:**
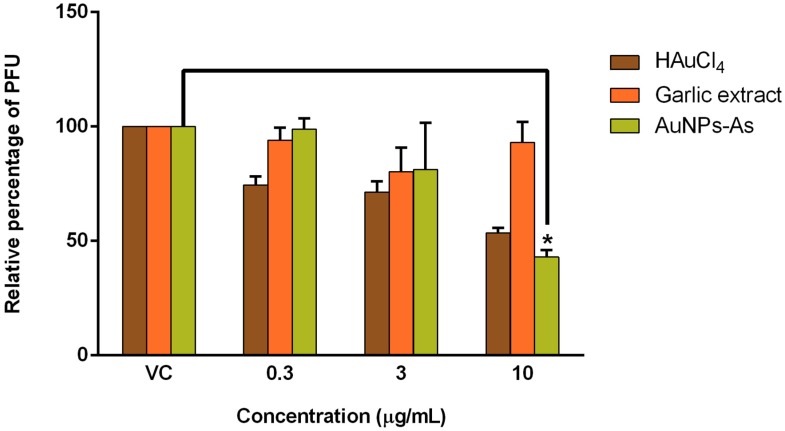
Antiviral activity of AuNPs-As, HAuCl_4_, and garlic extract against MeV (Plaque Formation Units (PFU) reduction). Vero cells (3 × 10^5^ cells) plated in 6-well plates were infected with Measles virus (MeV) and treated with different concentrations of AuNPs-As during and after infection for 72 h, and the antiviral activity was determined in a PFU reduction assay. The data are expressed as relative PFU (%) to that for the untreated virus-infected control cells (Viral control), which was defined as 100%. The data shown are the mean ± SD from three replicated experiments. α ≤ 0.05 (*).

AuNPs-As and their precursors, HAuCl_4_ and garlic extract (*Allium sativum*) were tested against MeV by PFU assays. The concentration of AuNPs-As at which infectivity inhibited by 50% (EC_50_) was 8.829 μg/mL, obtaining an antiviral activity of 57.07% with the highest concentration of 10 μg/mL (Figure 3). To define whether the antiviral effect obtained is due to nanoparticles, rather than their precursors, we assessed the antiviral activity of the precursor HAuCl_4_ and garlic extract, obtaining an antiviral activity of 46.43% and 6.96%, respectively, with the highest concentration of 10 μg/mL (Figure 3). We also evaluated the antiviral activity of AuNPs-As with the non-cytotoxic concentrations of 0.3 and 3 μg/mL; the PFU assays, however, did not show significant antiviral activity at the highest concentration used (3 μg/mL), reaching 21.24% of PFU inhibition [13].

As shown in Table 1, the selectivity index (SI) of each treatment was calculated. The SI reflects a compound’s overall activity by relating cytotoxicity (CC_50_) and effectiveness, measured as the ability to inhibit infection (EC_50_). SI calculation of AuNPs-As and HAuCl_4_ was 16.05 and 7.4, respectively. AuNPs-As showed the best SI values and, therefore, was selected for the subsequent experiments.

### 3.4. Effect of AuNPs-As on Viral Infection Was Determined by Time of Addition Assays

**Figure 4:**
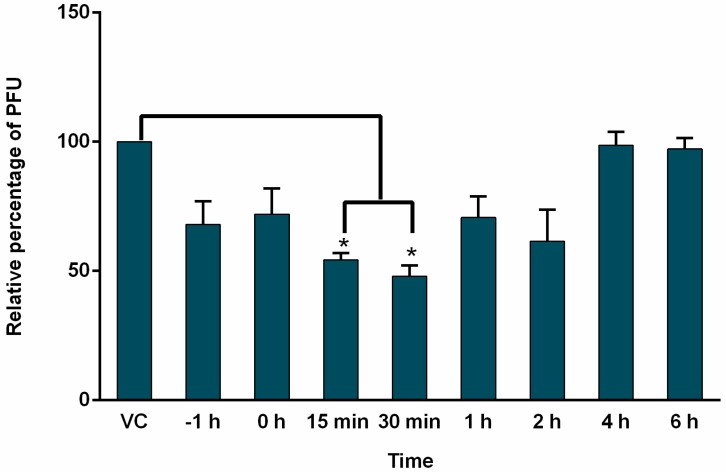
Time of addition experiments. Vero cells (3 × 10^5^ cells) plated in 6-well plates were infected with MeV, treated with AuNPs-As at different times of infection and analyzed by PFU inhibition assays. AuNPs-As was added at 1 h pre-infection and 0, 15, 30 min, 1, 2, 4 and 6 h post-infection. The data are expressed as relative PFU (%) compared to that of untreated virus-infected control cells (Viral control), which was defined as 100%. The data shown are the mean ± SD of triplicate experiments. α < 0.05 (*).

To determine which step of the MeV cycle was targeted by AuNPs-As, “time of addition” experiments were performed. Vero cells were infected and exposed to AuNPs-As (EC_50_ = 8.829 μg/mL) at different times of infection. As shown in Figure 4, the most efficient inhibition was observed in early steps of infection (15 and 30 min after infection). 

### 3.5. Virucidal Effect of AuNPs-As

**Figure 5:**
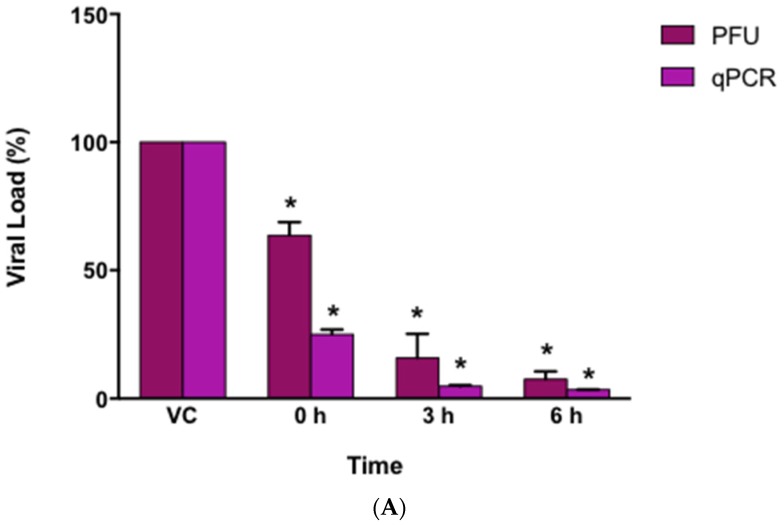

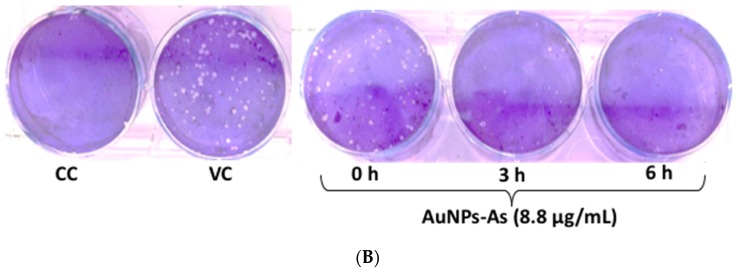
Virucidal effects of AuNPs-As. (**A**) Vero cells were infected with MeV previously exposed to AuNPs-As at different times. The virucidal effect was determined by PFU reduction and qPCR assays. PFU count and viral RNA number are given in % to that for the untreated virus-infected control cells (Viral control), which was defined as 100%. (**B**) Representative images of the virucidal effect of AuNPs-As, showing cellular control (CC), viral control (VC), and treatment at different periods of incubation (0, 3, 6 h). The data shown are the mean ± SD from three replicated experiments. α ≤ 0.05 (*) compared vs viral control.

Virucidal effect tests were performed by the technique of PFUs and qPCR to determine if the AuNPs-As had an effect directly on the viral particle by preventing its contact with the cell. AuNPs-As (EC_50_ = 8.829 μg/mL) were mixed with the virus during different times (0, 3, and 6 h) before infection, to evaluate the percentage reduction of PFUs or viral load. As shown in Figure 5A, AuNPs-As exhibited a reduction of 84% of PFU at 3 h of incubation and 92% at 6 h of incubation with the EC_50_ of the virus. When measuring the viral load by qPCR, a reduction of 95% was obtained at 3 h and 97% at 6 h of incubation. 

**Figure 6:**
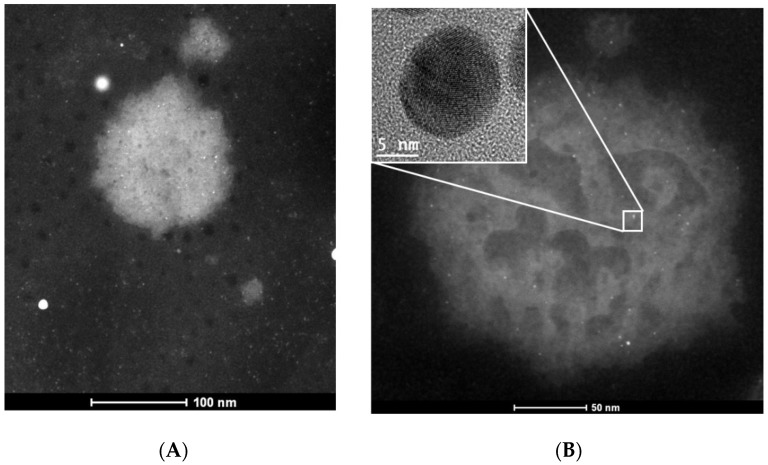
TEM images of the measles virus. (**A**) TEM image of a Measles virus. (**B**) TEM image of a measles virus exposed to AuNPs-As. Inset shows the nanoparticle that is interacting with the measles virus envelope.

HAADF-STEM images obtained from these suspensions corroborate the AuNPs-As/viral interactions (Figure 6).

**Figure 7:**
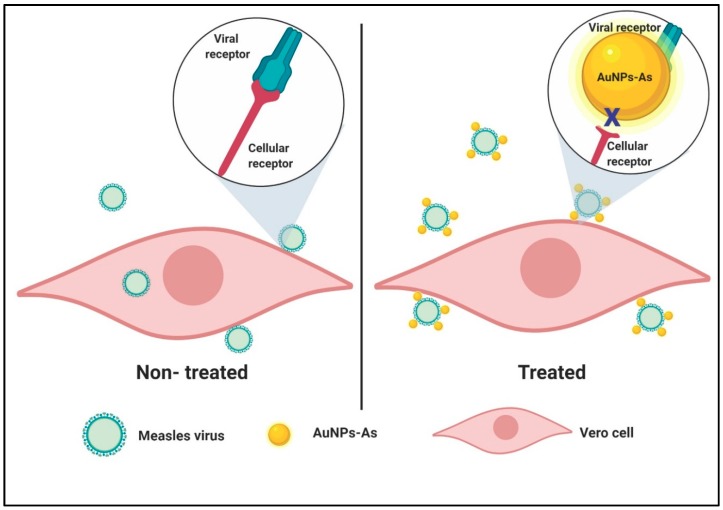
Schematic representation of a proposed mode of virucidal effect of AuNPs-As on Measles virus infection. Viral adsorption is started once the virion reaches Vero cells and the infection can be achieved successfully (left panel). In cells treated with AuNPs-As, the adsorption step is blocked by the binding of AuNPs-As and the viral envelope, therefore, the infection cannot be initiated (right panel). Created with BioRender.com.

Accordingly, we propose that AuNPs-As bind to the viral particle blocking the union with cell receptors inhibiting the start of the viral cycle (see Figure 7).

## 4. Discussion

Current antivirals compounds have limited effects against a select group of viruses, specifically, for MeV there is no a therapeutic alternative [5]. The bioactive composites extracted of natural sources have shown antiviral properties and high chemical reactivity which confers the reductive capacity of metallic salts as AgNO_3_ and HAuCl_4_ [6]. The formation of NPs by green chemistry provides a low cost, environment friendly technique that is easy to apply for large scale [7,14].

In this study we tested the antiviral activity of synthetized gold nanoparticles using garlic extract (As) as reducing agent. Figure 1A shows a characteristic resonance plasmon at 537 nm AuNPs-As, this data corresponds to previous reports in the literature. Rastogi and Arunachalam [15] reported that plasmon resonance must be between 500 and 600 nm absorbance; these authors also described the superficial plasmon resonance of gold nanoparticles synthesized with garlic extract to be of 520 nm. The particle sizes obtained by DLS (Figure 1B) were confirmed by Transmission Electron Microscopy (TEM) (Figure 1D), which supported the presence of smooth surface nanoparticles and spherical morphology in the suspension. The Z potential obtained from AuNPs-As was −92.5 and 21.2 mV (Figure 1C). Farooq et al. [16] reported that a Z potential of −90 is characteristic of pure H_2_O at pH 9 and that the potential becomes more negative as the alkalinity of the water increases. This data supports the results obtained, considering that the garlic extract used in the synthesis was previously alkalinized to a pH of 9 to avoid the precipitation of NPs in the medium. This justifies that the negative Z potential obtained was due to the garlic extract, demonstrating that the surface charge of the nanoparticles is positive (21.2 mV). The Z potential is an important physical-chemical parameter that influences the stability of nano suspensions. An extremely positive or negative Zeta potential value produces higher repulsive forces, and repulsion between particles with similar electrostatic charges prevents the aggregation of the particles, ensuring easy redispersion [17,18]. In the case of electrostatic and steric stabilization, a Z potential of ± 20 mV is adequate [19]; considering this, the stability of the synthesized nanoparticles can be confirmed. 

As shown in Figure 2, cytotoxic assays described that Vero cells treated with up to 100 µg/mL of gold nanoparticles with garlic extract (AuNPs-As) were not significantly different from non-treated control. When the cells were treated with an increased nanoparticle concentration (200 μg/mL), cytotoxicity was observed; cell survivability decreased by 90%. However, HAuCl_4_ by itself also showed similar results. Therefore, these cytotoxicity results could have been due to this precursor rather than the particles themselves. Low cytotoxicity is one of the most important requirements for any potential antiviral agent; Fatima et al. described the non-toxicity (CC_50_ > 500 μg/mL) of synthesized green nanoparticles and their precursors tested in the same way by the MTT assay on Vero cells [20]. Cagno et al. also reported the lack of significant toxicity at similar concentrations (CC_50_ ≥ 100–300 μg/mL) of gold NPs coated with undecanesulfonic acid (MUS)-containing ligands and their pre-MUS ligand [21].

The antiviral activity of the AuNPs-As showed a reduction of PFU of 57.07% at 10 μg/mL (Figure 3) obtaining an EC_50_ of 8.829 μg/mL (Table 1). We also tested the antiviral activity of the precursor HAuCl_4_ and garlic extract, obtaining an antiviral activity of 46.43% and 6.96%, respectively, with the highest concentration of 10 μg/mL (Figure 3). From these results, we could conclude that the antiviral activity of AuNPs-As was better than their precursors. Therefore, AuNPs-As might be an excellent virus inactivating agent against MeV infection. AuNPs-As showed a SI of 16.05 (Table 1), it is important to know the SI of a possible antiviral compound as it is a measure of the safety margin of a drug. It is expressed numerically as a ratio between the dose of the drug that causes death (CC_50_) and the dose causing the desired therapeutic effect (EC_50_). The safety is lower if the index value is lower, and therefore the consumption of the drug is very dangerous when the value approaches 1 [22]. There are very few drugs available for the treatment of infections caused by RNA viruses. The SI of the approved antiviral Ribavirin has been evaluated in vitro against several viruses of human importance obtaining SI of 0.1 for ZIKV, SI = 1 for WNV, SI = 3 for DENV-1, and SI = 11.5 for measles [23,24]. Therefore AuNPs-As showed higher efficacy against measles compared to that reported for ribavirin. To compare traditional synthesis with the green chemistry technique, we also evaluated the antiviral activity of AuNPs-Chi; however, we did not find antiviral activity at non-cytotoxic concentrations. Ahmed et al. tested the antiviral activity of ribavirin-AuNPs against MeV on Vero cells, they showed that ribavirin-AuNPs had a higher antiviral activity with lower dose (99.5 µg/mL) than ribavirin alone (500 µg/mL) and the maximal activity showed when it used after the virus infection [25]. AuNPs-As tested in this study were effective at a concentration 10 times lower than ribavirin-AuNPs tested by Ahmed et al. 

AuNPs-As are highly stable after synthesis and biocompatible and could easily interact with diverse bioactive compounds that conform the garlic extract, including organosulfur compounds, saponins, phenolic compounds, and polysaccharides. The mayor active components are its organosulfur, such as allicin and allicin-derived products (diallyl sulfide, diallyl disulfide, diallyl trisulfide, ajoene, allyl-cysteine, and allyl-cysteine sulfoxide) [26], which would confer them characteristics that could improve their biological activity [15]. This background highlights the safety of the use of AuNPs-As as a possible treatment against MeV. The antiviral activity of green synthesized nanoparticles has been mostly focused on the study of silver nanoparticles [27,28,29]. There is also limited information on gold nanoparticles with antiviral activity against enveloped viruses, however, Di Gianvincenzo et al. [30] synthesized and evaluated gold nanoparticles coated with multiple copies of an amphiphilic sulfate anti-HIV ligand. They tested different grades of sulfate density on the gold surface (100, 50, and 20%), and reported a significant viral reduction at a concentration of 100 µg/mL, with gold nanoparticles having a higher grade of sulfate density (100 and 50%).

To determine the action level of the AuNPs-As, time addition assays were performed, in which the effect of the NPs is evaluated when they are added to the cells at different times of infection. According to the results obtained (Figure 4), AuNPs-As exert an inhibitory effect from the time of pre-infection until 2 h after infection, observing a greater significant effect at 15 and 30 min. These results allow us to propose an effect in the first steps of the viral cycle, probably blocking cellular receptors or even blocking viral receptors (virucidal effect).

To assess the possible blockage of viral receptors by AuNPs-As, a virucidal assay was performed (Figure 5). The results (using EC_50_) showed a reduction of viral infection of 84% after 3 h interaction with the AuNPs-As and a reduction of 92% after 6 h of interaction (these results were confirmed by qPCR). There are currently some antecedents of metallic nanoparticles with virucidal effect. Mehrbrod et al. [31] evaluated the antiviral activity of silver nanoparticles against influenza virus, determining a reduction in Hemagglutination assay (HA) titre once NPs were added to infected cells. This effect was due to the previously demonstrated affinity of the NPs to the disulfide bridges that are present between the HAs, thus preventing the virus from binding to the receptor cell. In another study, Lara et al. [32] demonstrated the virucidal activity of PVP-coated silver NPs against HIV-1. Using ELISA assays, they confirmed the inhibition of gp120-CD4 binding and proposed a coupling of NPs to gp120 which, therefore, blocks interaction with CD4. Rai et al. [8] demonstrated that the virucidal potential of the NPs can be increased if the exact site of interaction and mode of action is known, leading to the modification of the surface of the NPs for a wider and more effective use. Recent advances in lipid mass spectrometry have made it possible to analyze the entire lipidoma of purified viruses. Quantitative analyzes of the lipid components of HIV revealed an enrichment of rafts of SM, cholesterol, and PE lipids with a high content of fatty acids in the viral membrane [33]. There are also reports of quantitative analysis of VSV and SFV lipidomas observing a similar composition between these and the plasma membrane from which their envelopes were derived [34]. Hashiguchi et al. reported the residues preserved in the hemagglutinin proteins (H) of seven morbilliviruses (measles, rinderpest, ruminant appetite plague, canine distemper, dolphin distemper, porpoise distemper, and fucina distemper) that interact with cellular receptors. They reported that the negatively charged cell receptor binding site in H proteins serves as the primary target for drugs/antibodies that block the early stages of MV infection [35]. 

Considering the previous studies and the results obtained regarding the positive surface charge of the AuNPs-As and the negative charge of the viral membranes (due to their lipidic composition and the negatively charged cell receptor-binding site), it is possible that the powerful virucide effect observed is due to the interaction of the NPs with the viral receptors. These findings were confirmed by TEM imaging where MeV was observed surrounded by AuNPs-As on the viral surface (Figure 6). Therefore, the interaction of the NPs and the MeV is likely to produce a blockage of the viral receptors, preventing cell adsorption and the onset of viral infection in the host cell (Figure 7). An “ideal” antiviral agent would precisely target the virus without interacting with host cells, so the virucidal effect of AuNPs-As offers a promising antiviral alternative against measles and viruses belonging to, or related to, the *Paramyxoviridae* family.

## 5. Conclusions

In this study, we tested the antiviral activity of gold nanoparticles synthesized by green chemistry using garlic extract (AuNPs-As) against Measles virus infection. The lack of cytotoxicity at inhibitory concentrations, as indicated by the selectivity index, demonstrated that AuNPs-As might be an excellent virus inactivating agent against MeV infection. Virucidal activity is not only a prophylactic strategy before viral infection but can also be successful as a treatment after infection, thereby avoiding virus dissemination. AuNPs-As may be useful as a promising strategy to treat and control the infection of MeV and other related enveloped viruses.

## Figures and Tables

**Table 1:** Selectivity index of AuNPs-As in Vero cell line Measles virus infected.

Treatment	CC_50_ (μg/mL)	CE_50_ (μg/mL)	SI
AuNPs-As	141.75	8.829	16.05
HAuCl_4_	231.74	31.4	7.4
Garlic extract	>1500	Undetermined	Undetermined
